# *Citrus reticulatae pericarpium* Extract Decreases the Susceptibility to HFD-Induced Glycolipid Metabolism Disorder in Mice Exposed to Azithromycin in Early Life

**DOI:** 10.3389/fimmu.2021.774433

**Published:** 2021-11-19

**Authors:** Hanqi Lu, Yanting You, Xinghong Zhou, Qiuxing He, Ming Wang, Liqian Chen, Lin Zhou, Xiaomin Sun, Yanyan Liu, Pingping Jiang, Jiaojiao Dai, Xiuqiong Fu, Hiu Yee Kwan, Xiaoshan Zhao, Linjie Lou

**Affiliations:** ^1^ Department of Traditional Chinese Medicine, Zhujiang Hospital of Southern Medical University, Guangzhou, China; ^2^ School of Chinese Medicine, Southern Medical University, Guangzhou, China; ^3^ Endocrinology Department, Nanfang Hospital, Southern Medical University, Guangzhou, China; ^4^ School of Chinese Medicine, Hong Kong Baptist University, Hong Kong, Hong Kong SAR, China; ^5^ The First Affiliated Hospital of Wenzhou Medical University, Wenzhou, China

**Keywords:** *Citrus reticulata pericarpium extract*, early life, antibiotic exposure, glycolipid metabolism disorder, TMAO

## Abstract

**Background:**

Studies have shown that gut microbe disorder in mice due to early-life antibiotic exposure promotes glycolipid metabolism disorder in adulthood. However, the underlying mechanism remains unclear and there is not yet an effective intervention or treatment for this process.

**Purpose:**

The study investigated whether early-life azithromycin (AZT) exposure in mice could promote high-fat diet (HFD)-induced glycolipid metabolism disorder in adulthood. Moreover, the effect of citrus reticulata pericarpium (CRP) extract on glycolipid metabolism disorder *via* regulation of gut microbiome in mice exposed to antibodies early in life were investigated.

**Methods and Results:**

Three-week-old mice were treated with AZT (50 mg/kg/day) *via* drinking water for two weeks and then were fed a CRP diet (1% CRP extract) for four weeks and an HFD for five weeks. The results showed that early-life AZT exposure promoted HFD-induced glycolipid metabolism disorder, increased the levels of inflammatory factors, promoted the flora metabolism product trimethylamine N-oxide (TMAO), and induced microbial disorder in adult mice. Importantly, CRP extract mitigated these effects.

**Conclusion:**

Taken together, these findings suggest that early-life AZT exposure increases the susceptibility to HFD-induced glycolipid metabolism disorder in adult mice, and CRP extract can decrease this susceptibility by regulating gut microbiome.

**Graphical Abstract:**
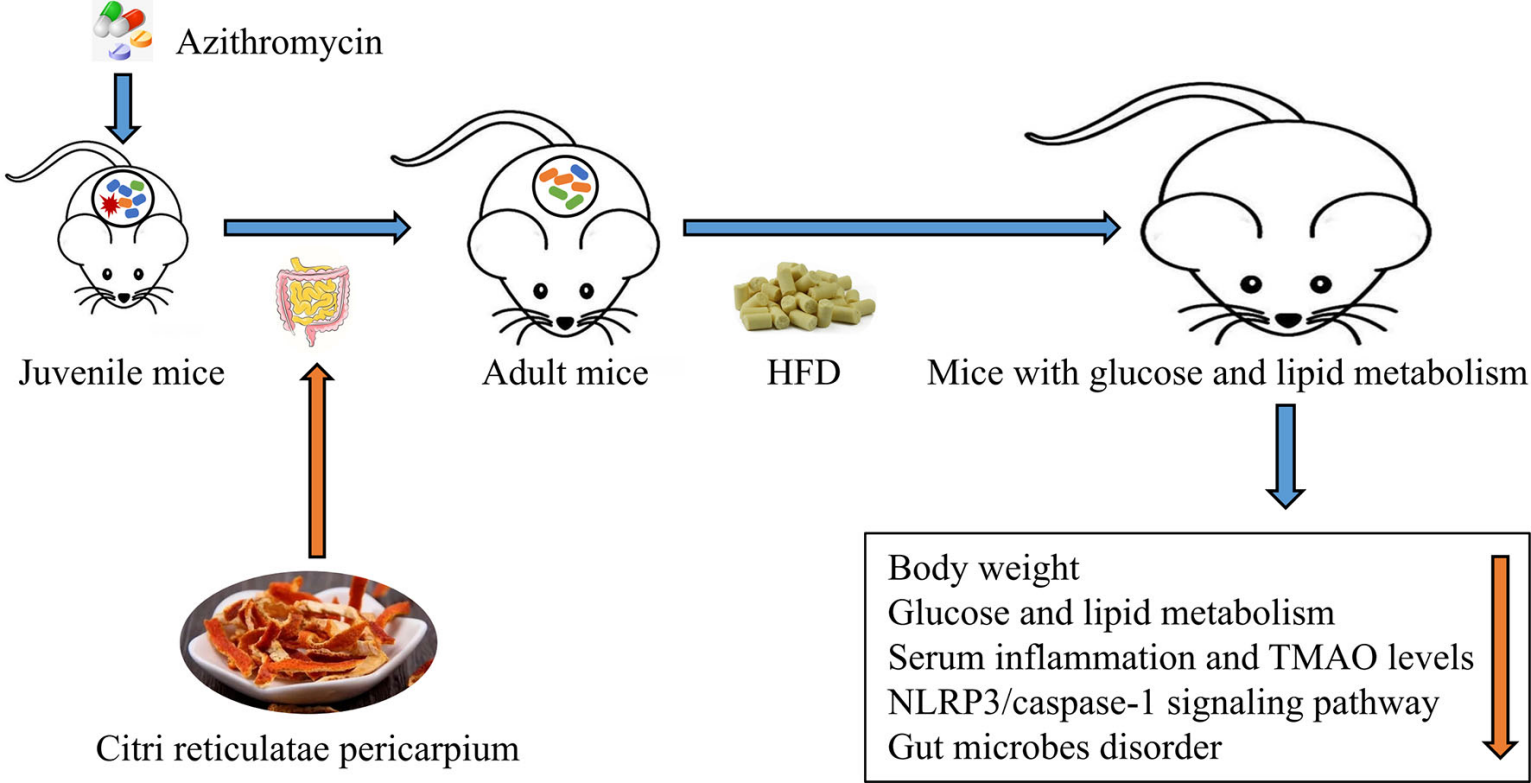
*Citrus reticulatae pericarpium* extract regulates AZT-induced gut microbial disorder in juvenile mice and reduces the level of the flora metabolism product TMAO under HFD feeding in adulthood, thereby reducing inflammation levels and improving glycolipid metabolism.

## Introduction

Glycolipid metabolism disorder, an important risk factor for cardiovascular diseases, is the primary feature of several metabolic diseases such as obesity, type 2 diabetes and non-alcoholic fatty liver. In China, the prevalence of obesity increased from 5.7% in 2010 to 6.3% in 2017, and the prevalence of diabetes increased from 9.7% in 2007 to 11.2% in 2017 (1). Abnormal glucose and lipid metabolism often occur in parallel. Therefore, preventing or reducing glycolipid metabolism disorder is of great practical significance.

Gut microbiome play an important role in the development of glycolipid metabolism disorder and many factors, such as the use of antibiotics, can lead to the disorder of gut microbiome (2). Antibiotic exposure in childhood changes the composition of the gut microbiome, leading to a decreased abundance of beneficial bacteria and an increased abundance of harmful bacteria; this then increases the susceptibility to glycolipid metabolism disorder in adulthood (3). Animal studies have shown that disorder of the gut microbiome in mice in early life can promote high-fat diet (HFD)-induced glycolipid metabolism disorder in adulthood (4, 5). However, the specific mechanism remains to be elucidated. Therefore, it is particularly important to further explore this mechanism and identify effective intervention measures.

Glycolipid metabolism disorder is accompanied by chronic low-level inflammation throughout the body; this is mainly manifested as increased expression levels of inflammatory factors such as IL-1β, IL-6 and TNF-α (6). The NLRP3/caspase-1 signalling pathway plays an important role in this process (7). NLRP3 inflammasomes are protein complexes composed of NLRP3, ASC and caspase-1. When NLRP3 inflammasomes are activated, NLRP3 and ASC form a complex, which activates caspase-1 to promote the maturation and release of IL-1β and IL-18 (8). Trimethylamine N-oxide (TMAO) is a metabolite in the gut; its expression level is positively correlated with body weight, blood sugar and other indicators (9). Studies have indicated that TMAO can activate NLRP3 inflammasomes, increase the body’s inflammation level, and promote abnormal glucose and lipid metabolism (10). Therefore, regulating the gut microbiome and reducing the production of TMAO, a metabolite of the gut, may be an important mechanism for improving glycolipid metabolism disorder.

Recently, Chinese medicines have been used to regulate the gut microbiome, and most have been found to improve glycolipid metabolism disorder (11, 12). *Citrus reticulatae pericarpium* (CRP), also known as chenpi, is the dry, mature peel of Citrus Reticulata Blanco (Rutaceae). CRP has long been used in traditional medicine for treating digestive tract diseases and anti-inflammatory diseases; it is also used as a seasoning in cooking and as a dietary supplement (13). Studies have shown that its extracts and active ingredients may improve glycolipid metabolism disorder by regulating the gut microbiome (14–17). Therefore, CRP extract was selected as an intervention drug in this study and its possible mechanism in glycolipid metabolism disorder was explored in juvenile C57BL/6 mice referred to existing researches (4, 5). Based on the literature, it was hypothesised that early life AZT exposure in mice could promote HFD-induced disorder of gut microbiome, increase the level of TMAO, increase the levels of inflammatory factors, and induce glycolipid metabolism disorder in adulthood. Moreover, it was predicted that CRP extract could improve this process by regulating AZT-induced disorder of gut microbiome in mice.

## Materials and Methods

### Materials

CRP granules were purchased from China Resources Sanjiu Pharmaceutical Co., Ltd (Guangzhou, Guangdong, China; Lot Number: 1706002S). AZT was purchased from Aladdin Bio-Chem Technology Co., Ltd (Shanghai, China). The production method for the CRP granules was as follows. First, the CRP extract was obtained by heating the pre-treated CRP twice. Then, the extracts obtained from each extraction process were mixed, filtered, and concentrated under reduced pressure into mushy extracts with a specific gravity of 1.20 to 1.35, respectively. Finally, the mushy extracts were spray-dried into granules. The normal diet (ND; D12450B) and high-fat diet (HFD; D12492) were purchased from the Guangdong Medical Laboratory Animal Centre (GDMLAC; Foshan, Guangdong, China). The specific ingredients of these diets are shown in **Table S1**. The CRP diet (1% CRP granules based on D12450B) was provided by GDMLAC (Foshan, Guangdong, China).

### UPLC-Q/TOF MS Analysis of CRP

UPLC-Q/TOF MS analysis of CRP was conducted to explore the possible effective ingredients of CRP and compare with the drug standard of CRP extract. The CRP extract (1 g of CRP granules dissolved in 10 ml pure water) was left to stand for 30 min, heated to reflux for 2 h, and then passed through a 0.22 m filter membrane for UPLC-QTOF-MS analysis. Separation was performed on a Waters XSelect HSS T3 (2.1 mm × 100 mm, 1.8 μm) column and elution was performed with mobile phases of 0.1% formic acid (A) and acetonitrile in water (B) in gradient mode. The proportion of acetonitrile varied from 10 to 90% in 32 min (0-8 min, 90-85% A, 10-15% B; 8-18 min, 85-70% A, 15-30% B; 18-28 min, 70-50% A, 30-50% B; 28-32 min, 50-10% A, 50-90% B) at a flow rate of 0.3 ml/min; each injection volume was set to 10 μl.

The scan time was 0.2 s (first level) and 0.1 s (second level). The acquisition time was 32 min. The acquisition range was 50-1500 Da. The atomization gas flow rate was 50 mL/min. The desolvent gas flow rate was 50 mL/min. The curtain gas flow rate was 35 mL/min. The desolventizing gas temperature was 500°C. The ion spray voltage was 4500 V (negative mode) and 5500 V (positive mode). The declustering voltage was 100 V. The collision energy was 10 V (first level) and 40 V (second level). The dynamic background subtraction mode was used. The mass spectrum drift range was 50 mDa. For analysis, 5 μl of the CRP extract was accurately drawn and detected by UPLC-Q/TOF MS.

### Animals and Treatment

**Figure 1:**
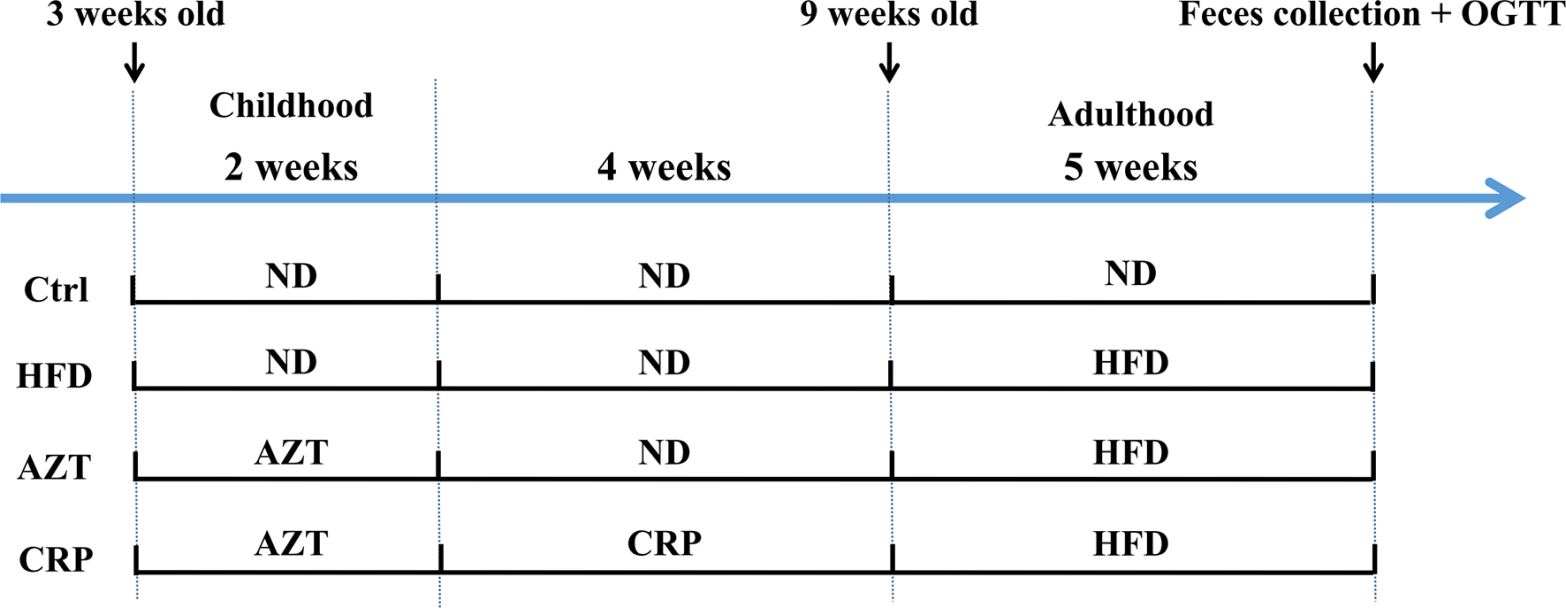
The experimental flow chart. Forty three-week-old mice were treated with or without AZT in drinking water for two weeks and were subsequently fed with or without a CRP diet for four weeks and then an ND or HFD for another five weeks.

The animal models were established with reference to existing researches (4, 5). Forty juvenile specific pathogen free (SPF) C57BL/6 mice (male, three-weeks-old, weight 12 ± 3g) were purchased from the GDMLAC (Permit number: SCXK 2013-0002). The animals were housed under standard laboratory conditions (22 ± 0.5°C, 40-70% relative humidity, and 12 h/12 h light/dark cycle), with a standard diet and water at libitum for three days. This study was carried out in accordance with the National Act on the Use of Experimental Animals (China). The estimated required sample size based on the degrees of freedom for analysis of variance was five in each group, but due to the large individual differences in the detection of gut microbiome, the sample size of each group was increased to ten in each group. The experimental grouping and procedures are shown in **Figure 1**. After three days of adaptation, the 40 mice were randomly divided into four groups (block random grouping, n=10, 2 cages, 5 in a cage): (1) Control (Ctrl) group: mice were fed the ND during the entire experimental process; (2) HFD group: mice were fed the HFD for five weeks starting in the 7th week of the experiment; (3) AZT group: mice were treated with AZT (50 mg/kg/day, conversion based on the body surface area of humans and mice) in the drinking water for two weeks at the beginning of the experiment and were fed the HFD for five weeks starting from the 7th week of the experiment; (4) CRP group: mice were treated AZT in drinking water for two weeks at the beginning of the experiment (as above) and were subsequently fed the CRP diet for four weeks and then the HFD for another five weeks. Throughout the experiment, the body weights of the mice were recorded weekly. The water tubes were replaced daily for the administration of antibiotics during AZT treatment. At the end of the experiment, the oral glucose tolerance test (OGTT) was performed, and stool samples were collected and stored at -80°C for further analyses. The mice were sacrificed after a 12 h fast. Blood was collected and rapidly centrifuged at 3000 rpm for 10 min at 4°C. Then, the serum was collected from the supernatant and stored at -20°C for subsequent analysis. Liver and abdominal adipose tissues were removed and weighed immediately. Some of the liver and adipose tissues were stored in 4% paraformaldehyde for pathological analysis, and the remaining tissues were stored at -80°C for further analysis.

### Oral Glucose Tolerance Test (OGTT)

The mice were fasted for 6 h before the experiment. The fasted mice were oral-gavaged with 20% (w/v) D-glucose solution (2 g/kg body weight; Sigma-Aldrich, USA) and tail vein blood was collected 0, 30, 60, 90 and 120 min after glucose gavage. The blood glucose level was measured using a blood glucose meter (Sano Biosensor Co., Ltd., Guangzhou, Guangdong, China). The area under the curve (AUC) was calculated to quantify the cumulative changes in the blood glucose response.

### Biochemical Analysis

Serum triglycerides (TG), total cholesterol (TC), low-density lipoprotein-cholesterol (LDL-C) and high-density lipoprotein-cholesterol (HDL-C) were determined using spectrophotometry, according to the manufacturer’s instructions (Jiancheng, Nanjing, China). The levels of TNF-α, IL-6, IL-1β (Beijing Solibao Technology Co., Ltd, China) and TMAO (Guangzhou Laizhi Biological Technology Co., Ltd, Guangzhou, Guangdong, China) in the serum were quantified using mice ELISA kits, according to the manufacturer’s instructions.

### Liver and Abdominal Adipose Tissue Histology

Mice liver and adipose tissues were fixed in 4% paraformaldehyde and embedded in paraffin. Next, the samples were sliced, stained with the haematoxylin and eosin (HE) method, and then observed under an optical microscope.

### Western Blotting Analysis

Western blot analysis of liver tissues was performed according to standard procedures using specific antibodies including NLRP3, caspase-1, IL-1β and IL-18 (Affinity Biosciences, USA). β-actin was used as an internal control. After reacting with the secondary antibody, proteins were detected with an enhanced chemiluminescence (ECL) Western blotting detection reagent (Millipore, USA) and visualised on a FluorChem E ultra-sensitive automatic imaging analysis system (ProteinSimple, USA).

### DNA Extraction and 16S rRNA Sequencing

The V3-V4 regions of 16S rRNA were amplified with the following primers: 314F: ACTCCTACGGGAGGCAGCAG; 805R: GGACTACHVGGGTWTCTAAT. The samples were sequenced on a HiSeq2500 PE250 (Illumina, Inc., USA). Analysis was performed at the phylum and genus levels. In-house Perl scripts were used to analyse alpha (within samples) and beta (among samples) diversity. The Shannon index was used to analyse alpha diversity. Principal coordinate analysis (PCoA) based on weighted UniFrac distance matrices was performed for the beta diversity analysis. Phylum- and genus-level taxonomic distributions of the microbial communities, a heat map at the genus level, and Spearman correlation analysis were used to identify specific bacteria.

### Statistical Analysis

All statistical analyses were performed using SPSS version 20.0 software (SPSS, Chicago, IL, USA). The data are presented as means ± SEM. Analysis of variance (ANOVA) was used to test for group differences, with the Bonferroni correction for *post hoc* comparisons. P <0.05 was considered to be statistically significant.

## Results

### UPLC-Q/TOF MS Analysis of RP

**Figure 2:**
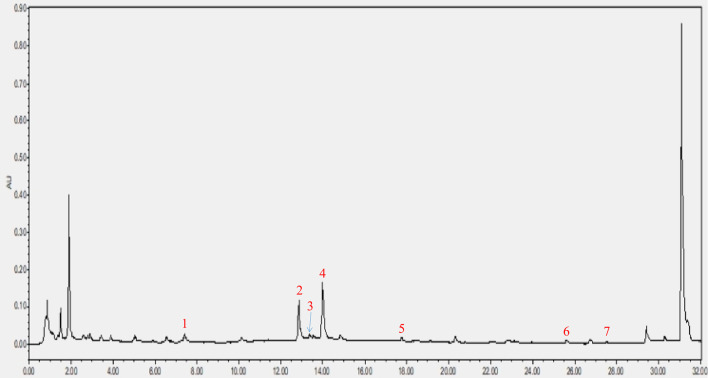
HPLC analysis of CRP extract. (1) narigin-4’-O-glucoside, (2) narirutin, (3) naringin, (4) hesperidin, (5) poncirin, (6) nobiletin and (7) tangeretin.

As shown in **Figure 2**, a total of seven components were identified in the CRP extract according to their retention times (**Table S2**).

### CRP Extract Reduced Body Weight in HFD-Induced Mice With Early-Life AZT Exposure

**Figure 3:**
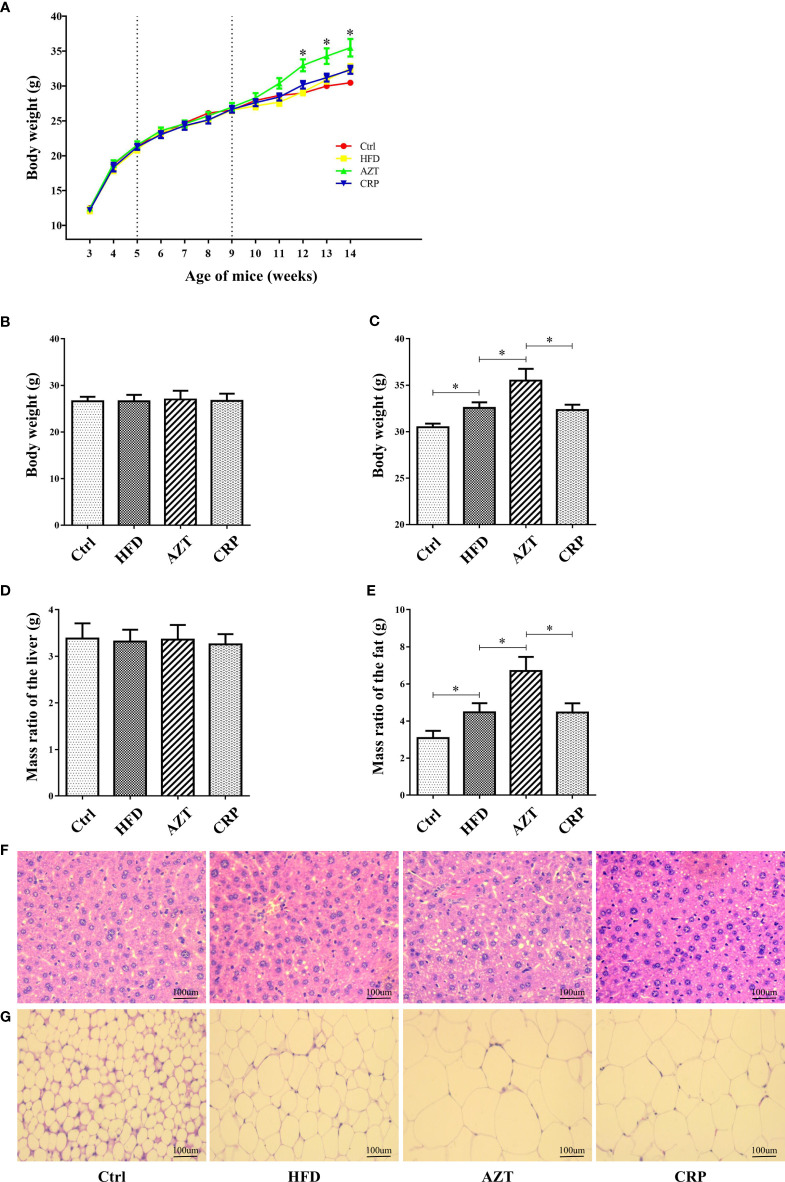
Phenotypic changes between the groups. **(A)** Changes in body weight throughout the experiment. **(B)** Changes in body weight in nine-week-old mice. **(C)** Changes in body weight in 14-week-old mice. **(D)** The ratio of adipose tissue to body weight. **(E)** The ratio of liver to body weight. **(F)** H&E staining of liver. **(G)** H&E staining of adipose tissue. (200×, scale bar, 100 µm). Differences were assessed by ANOVA. Data are expressed as the mean ± SEM, n = 10 in each group. *P < 0.05.

As expected, there was no significant difference in body weight between the groups before the 6th week of the experiment; differences were observed in the 7th week of the experiment (**Figures 3A, B**). At the end of the experiment, the body weight and the adipose tissue/body weight ratio in the HFD group were significantly higher than the Ctrl group. Notably, the AZT group exhibited a significantly increased body weight and adipose tissue/body weight ratio than the HFD group, while CRP significantly mitigated this increase in body weight and the adipose tissue/body weight ratio (**Figures 3C, D**). However, no significant difference was observed between the groups in the liver/body weight ratio (**Figure 3E**). Histological analysis showed that mice in the HFD group had more severe liver steatosis and bigger adipocyte size than the Ctrl group. Mice in the AZT group had more severe liver steatosis and bigger adipocyte size than the HFD group, and these histological changes were ameliorated in the CRP group (**Figures 3F, G**). These results suggest that CRP extract reduces body weight and modifies glycolipid metabolism disorder in HFD-induced glycolipid metabolism disorder mice treated with AZT.

### CRP Extract Improved Glycolipid Metabolism in HFD-Induced Mice With Early-Life AZT Exposure

**Figure 4:**
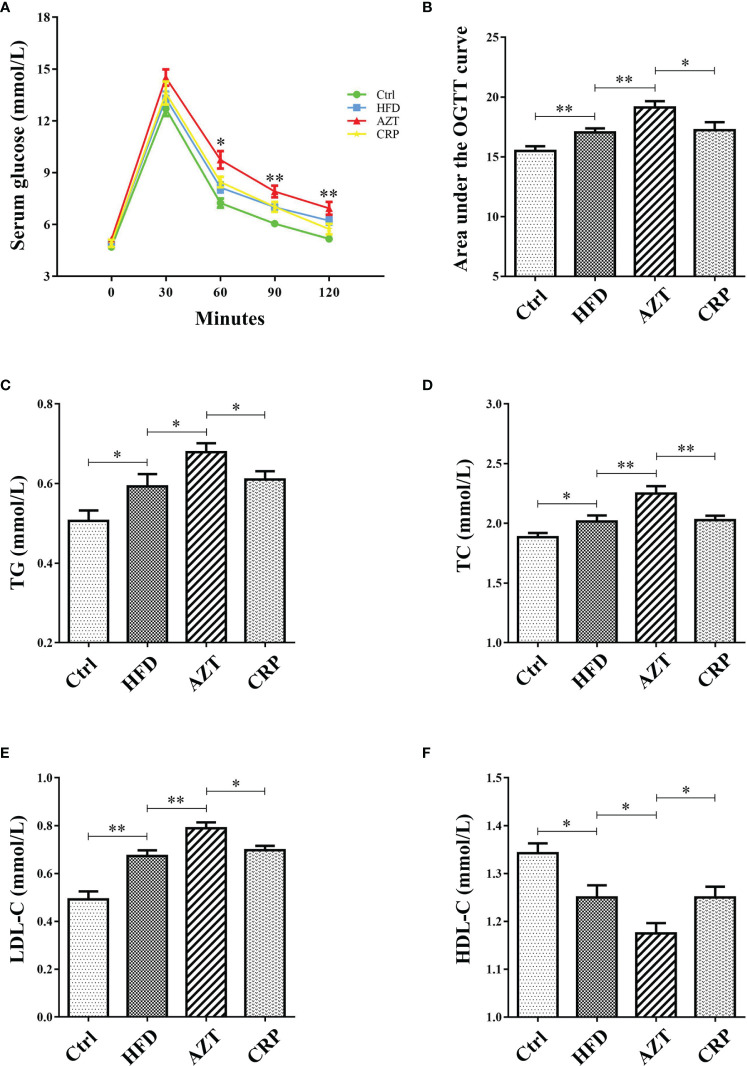
Indices of glycolipid metabolism between the groups. **(A)** OGTT curve. **(B)** Area under the curve (AUC) of the OGTT. **(C)** Serum triglycerides (TG). **(D)** Serum total cholesterol (TC). **(E)** Serum low-density lipoprotein-cholesterol (LDL-C). **(F)** Serum high-density lipoprotein-cholesterol (HDL-C). Differences were assessed by ANOVA. Data are expressed as the mean ± SEM, n = 10 in each group. *P < 0.05, **P < 0.01.

As expected, glucose tolerance in the HFD group was increased compared to the Ctrl group. More importantly, the AZT group showed increased glucose tolerance compared to the HFD group, and the CRP diet restored the glucose tolerance of the AZT-treated mice (**Figures 4A, B**). In addition, serum TG, TC and LDL-C levels were markedly increased, and HDL-C levels were significantly decreased in the HFD group compared to the Ctrl group. The AZT group exhibited further exacerbation of lipid metabolism disorder, and the CRP diet improved this disorder in the AZT-treated mice (**Figures 4C–F**). These results suggest that CRP extract improves glucose and lipid metabolism in HFD-induced glycolipid metabolism disorder mice treated with AZT

### CRP Extract Reduced Serum Inflammation Levels in HFD-Induced Mice With Early-Life AZT Exposure

**Figure 5:**
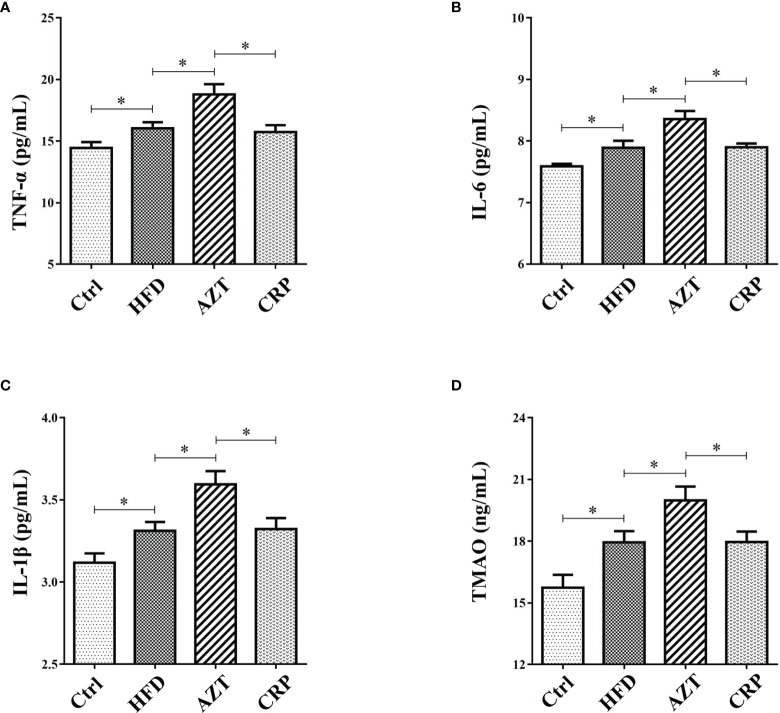
Serum inflammation and trimethylamine N-oxide (TMAO) levels between the groups. **(A)** Tumour necrosis factor-α (TNF-α). **(B)** Interleukin-6 (IL-6). **(C)** Interleukin-1β (IL-1β). **(D)** TMAO. Differences were assessed by ANOVA. Data are expressed as the mean ± SEM, n = 10 in each group. *P < 0.05.

The ELISA results showed that the serum levels of TNF-α, IL-6 and IL-1β in the HFD group were increased compared to the Ctrl group. Notably, AZT further increased the TNF-α, IL-6 and IL-1β levels compared to the HFD group, while the CRP diet mitigated these increases in TNF-α, IL-6 and IL-1β levels in the AZT-treated mice (**Figures 5A–C**). These results suggest that CRP extract reduces serum inflammation levels in HFD-induced glycolipid metabolism disorder mice treated with AZT.

### CRP Extract Reduced Serum TMAO Levels in HFD-Induced Mice With Early-Life AZT Exposure

The results showed that serum levels of TMAO in the HFD group were increased compared to the Ctrl group. More importantly, the AZT group exhibited a further increase in TMAO levels compared to the HFD group, and the CRP diet significantly decreased the serum TMAO levels compared to the AZT group (**Figure 5D**). These results suggest that CRP extract reduces serum TMAO levels in HFD-induced glycolipid metabolism disorder mice treated with AZT.

### CRP Extract Inhibited the NLRP3/Caspase-1 Signalling Pathway in HFD-Induced Mice With Early-Life AZT Exposure

**Figure 6:**
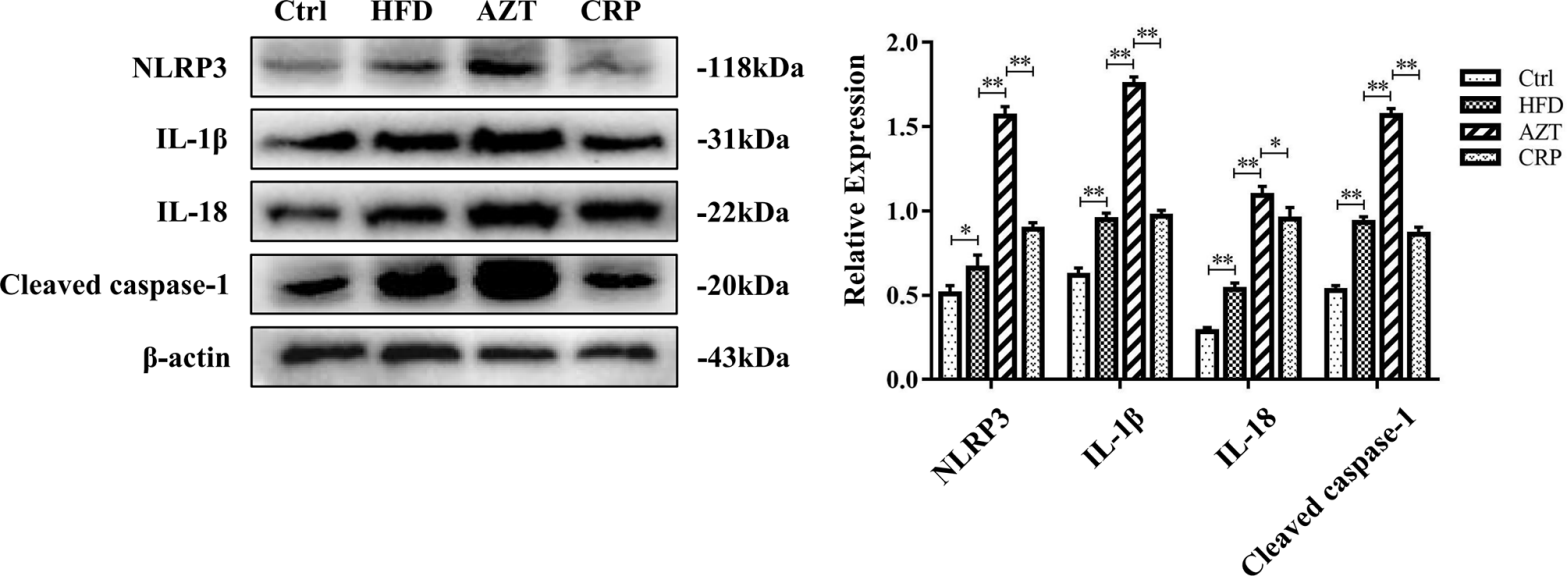
Expression levels of liver NLRP3/caspase-1 signalling pathway-related proteins and grey value analysis between the groups. Differences were assessed by ANOVA. Data are expressed as the mean ± SEM, *P < 0.05, **P < 0.01.

The Western blotting results showed that the expressions of the NLRP3, caspase-1, IL-1β and IL-18 proteins in the livers of the HFD group were increased compared to the Ctrl group. Notably, the AZT group exhibited further increased expression of the NLRP3, caspase-1, IL-1β and IL-18 proteins in the liver compared to the HFD group, and the CRP diet significantly reduced the expression of these liver proteins compared to the AZT group (**Figure 6**). These results suggest that CRP extract reduces the expression of liver NLRP3/caspase-1 signalling pathway-related proteins in HFD-induced glycolipid metabolism disorder mice treated with AZT.

### CRP Extract Recovered the Disordered Gut Microbiome in HFD-Induced Mice With Early-Life AZT Exposure

**Figure 7:**
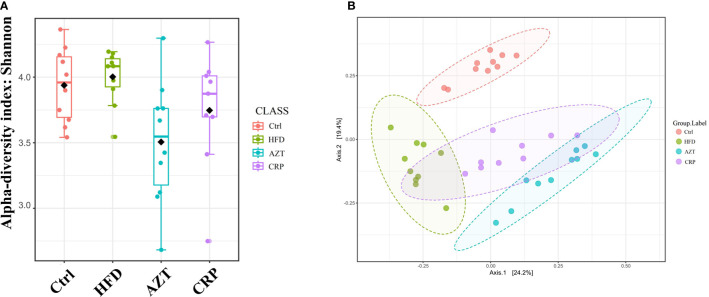
Alpha diversity and beta diversity between the groups (n = 9 for the CRP group and n = 10 for the other groups). **(A)** Alpha diversity. The larger the value, the higher the community richness of the gut microbe. **(B)** Beta diversity. The greater the distance between the groups, the greater the difference in community richness.

The Shannon index showed that the community richness in the guts of mice in the AZT group was significantly decreased compared to the HFD group, and the CRP group showed increased community richness compared to the AZT group. However, there was no significant difference in community richness between the HFD group and Ctrl group (**Figure 7A**). UniFrac-based PCoA revealed that the HFD, AZT and CRP groups clustered differently from the Ctrl group, and there were overlaps in these groups (**Figure 7B**).

**Figure 8:**
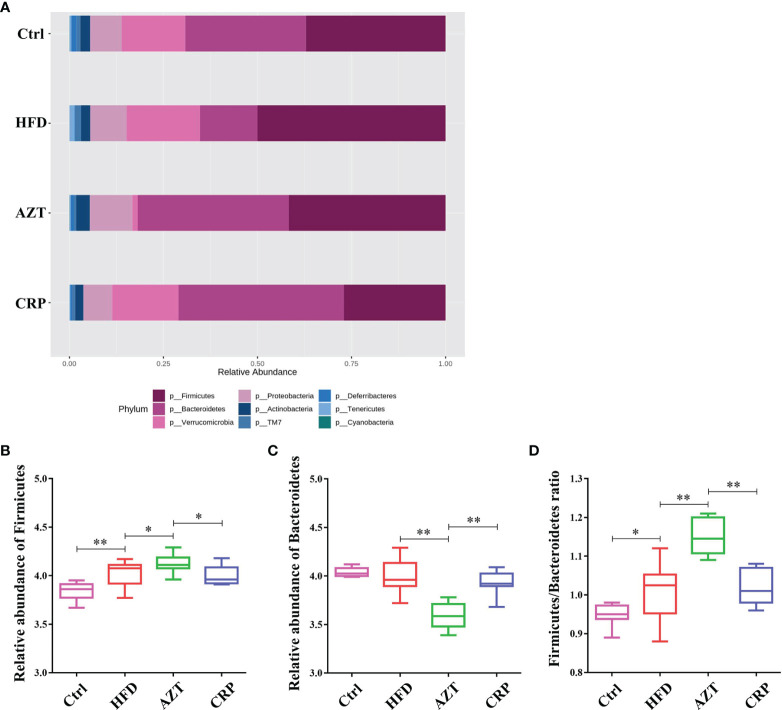
Changes in microbes at the phylum level in faeces between the groups. **(A)** Changes in the relative abundance of microbes at the phylum level. **(B)** Relative abundance of Firmicutes. **(C)** Relative abundance of Bacteroidetes. **(D)** Firmicutes/Bacteroidetes ratio. Differences were assessed by ANOVA. Data are expressed as the mean ± SEM, n = 9 for the CRP group and n = 10 for the other groups. *P < 0.05, **P < 0.01.

At the phylum level, Firmicutes and Bacteroidetes were the dominant phyla in these groups (**Figure 8A**). The HFD significantly increased the relative abundance of Firmicutes in the gut microbiome. However, there was no significant difference in the relative abundance of Firmicutes between the HFD group and Ctrl group. Notably, the AZT group showed increased relative abundance of Firmicutes and decreased relative abundance of Bacteroidetes compared to the HFD group. The CRP group showed significantly decreased relative abundance of Firmicutes and increased relative abundance of Bacteroidetes compared to the AZT group (**Figures 8B, C**). Overall, the ratio of Firmicutes/Bacteroidetes was increased in the AZT group and reduced in the CRP group (**Figure 8D**).

**Figure 9:**
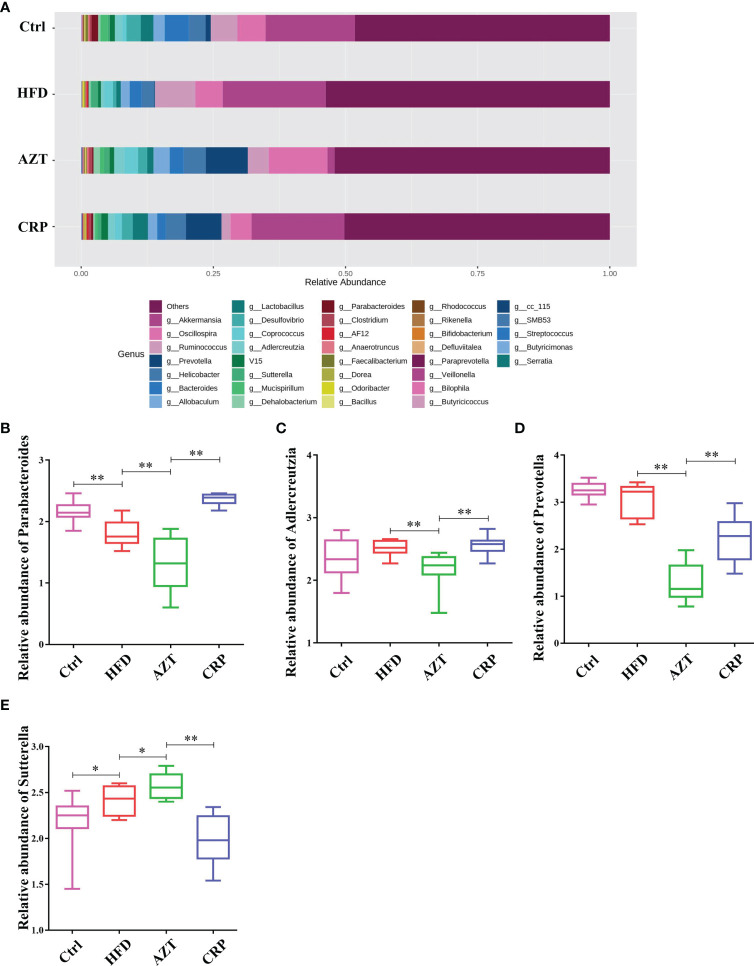
Changes in microbes at the genus level in faeces between the groups. **(A)** Changes in the relative abundance of microbes at the genus level. **(B)** Relative abundance of Parabacteroides. **(C)** Relative abundance of Adlercreutzia. **(D)** Relative abundance of Prevotella. **(E)** Relative abundance of Sutterella. Data are expressed as the mean ± SEM, n = 9 for the CRP group and n = 10 for the other groups. *P < 0.05, **P < 0.01.

At the genus level, the HFD group showed significantly decreased relative abundance of Parabacteroides and increased relative abundance of Sutterella compared to the Ctrl group. The AZT group showed significantly decreased relative abundance of Parabacteroides, Adlercreutzia and Prevotella, and increased relative abundance of Sutterella compared to the HFD group. More importantly, the CRP group showed significantly increased relative abundance of Parabacteroides, Adlercreutzia and Prevotella, and decreased relative abundance of Sutterella compared to the AZT group (**Figure 9**).

**Figure 10:**
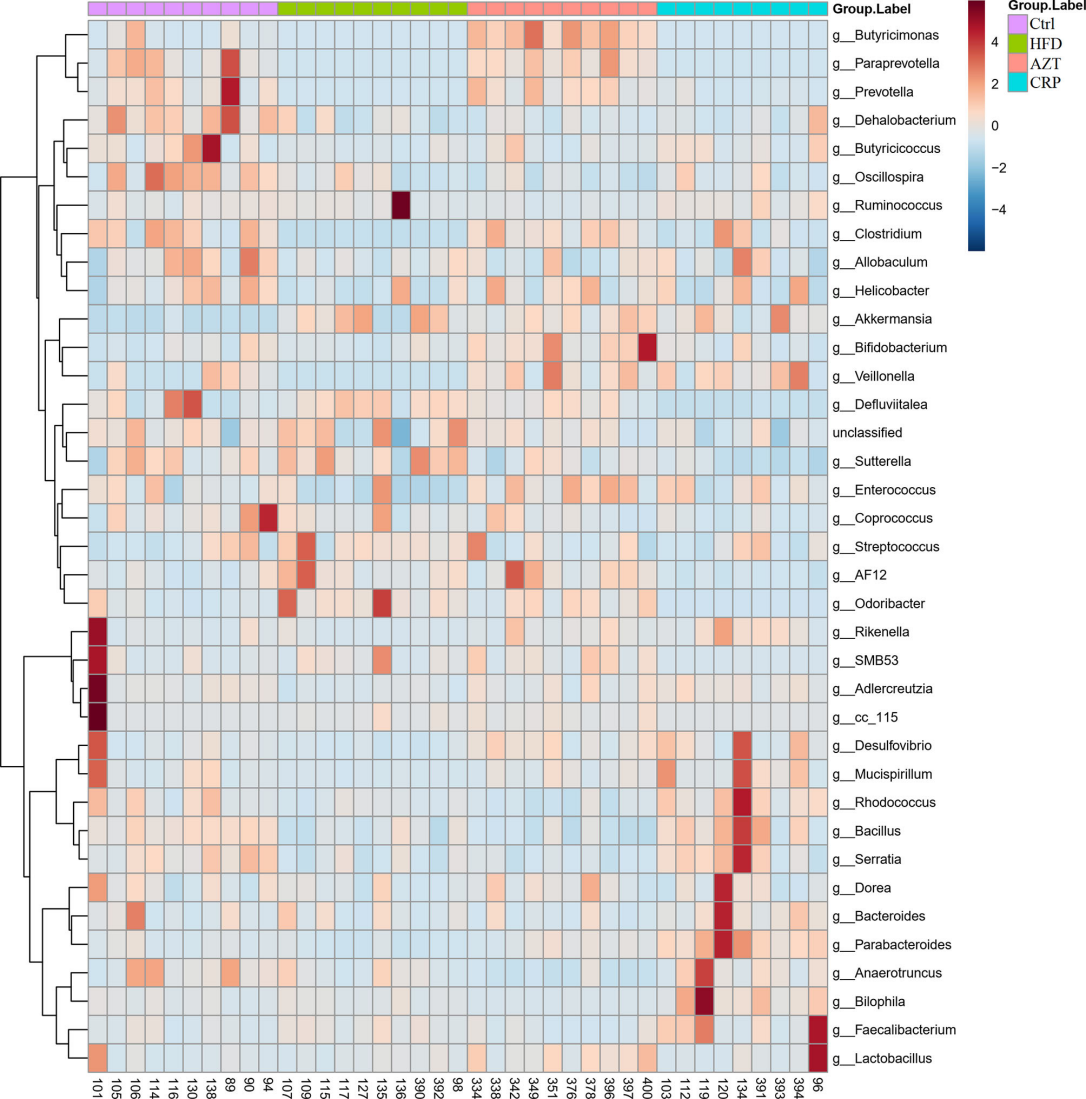
Heat map at the genus level between the groups. Red indicates high values; blue indicates low values.

**Figure 11:**
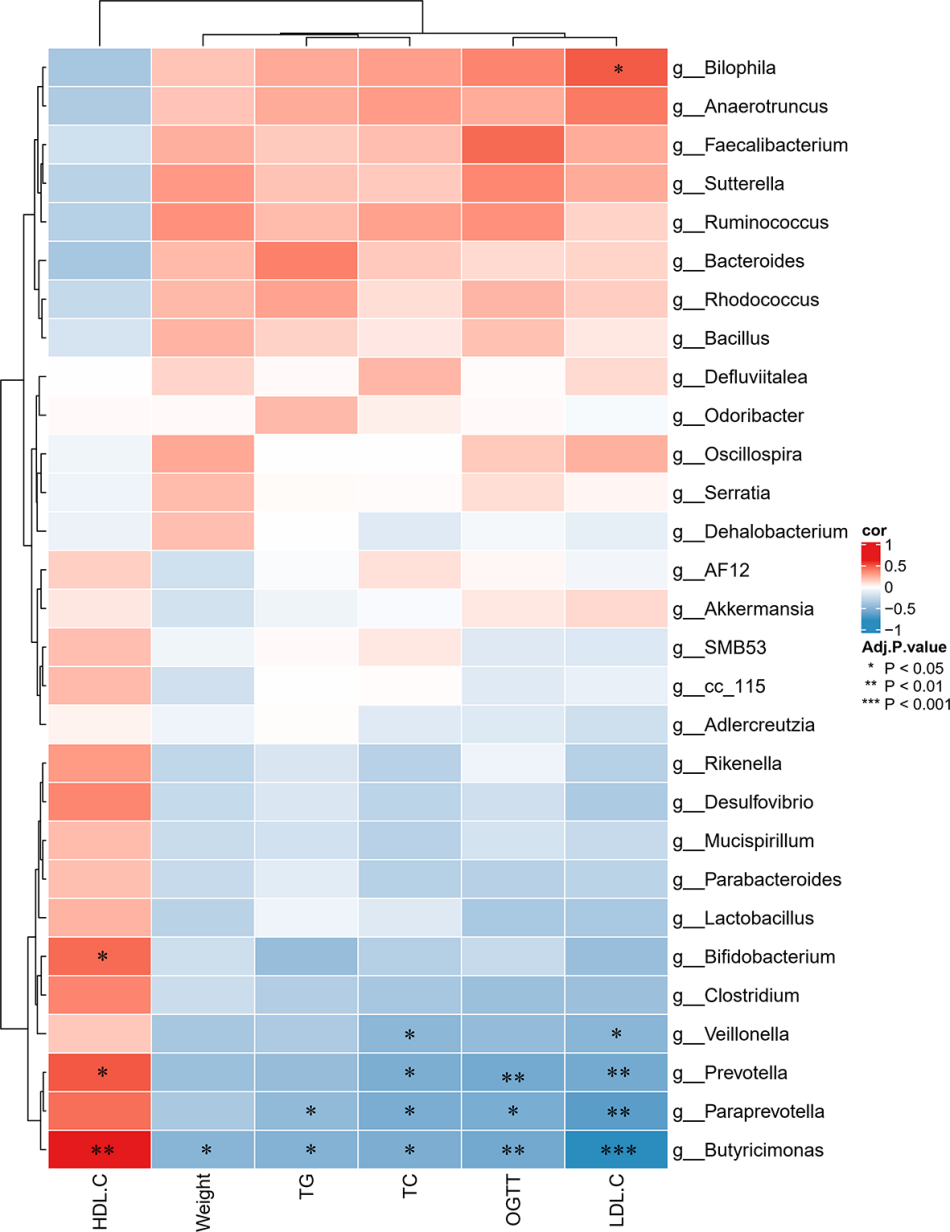
Spearman correlations between glycolipid metabolism indices and microbes at the genus level in the four groups of mice. Red indicates high values; blue indicates low values. Data are expressed as the mean ± SEM, n = 9 for the CRP group and n = 10 for the other groups. *P < 0.05, **P < 0.01, ***P < 0.001.

To further determine the differences in the faecal microbiota community between the groups, 36 genera are presented as a function of relative abundance in a heat map (see **Figure 10**). The relative abundance of certain bacteria, such as an increased relative abundance of Enterococcus and Streptococcus in the AZT group and a decreased relative abundance in the CRP group, can be seen in the heat map; however, there were no significant differences. Spearman correlation analysis revealed that improved body weight and glucose and lipid metabolism were negatively correlated with the relative abundance of Bilophila and positively correlated with the relative abundance of Bifidobacterium, Veillonella, Prevotella, Paraprevotella and Butyricimonas (**Figure 11**). Collectively, these results show that CRP extract improves gut microbiome disorder in HFD-induced glycolipid metabolism disorder mice treated with AZT.

## Discussion

AZT is a second-generation, broad-spectrum macrolide antibiotic. AZT is mainly used to treat respiratory infections in children; its use in the paediatric population is preceded only by penicillin (18). Although penicillin is the dominant antibiotic used in the paediatric population, studies have shown that the use of macrolide antibiotics causes more serious disorder of the gut microbiome than penicillin under the same conditions (3). Studies have shown that disorder of the gut microbiome caused by AZT exposure in juvenile mice can promote HFD-induced glycolipid metabolism disorder in adulthood (4, 5). Therefore, in this study, AZT exposure was first used to induce gut microbiome disorder in juvenile mice, and then these mice were fed an HFD in adulthood to induce glycolipid metabolism disorder. Finally, CRP extract was used to treat these mice to investigate whether CRP regulated the disordered gut microbiome in these mice.

CRP is widely used in medicines for the treatment of diseases and as a material for food preparation. Thus, there is a long history of evidence of its impact on the daily health of the whole people. Therefore, CRP extract, rather than a component of CRP extract, was chosen as a treatment, and its efficacy and possible mechanism of action were observed. In order to explore the possible components of CRP extract, UPLC-Q/TOF MS analysis was first performed. The results showed that among the seven components detected, naringin, hesperidin, poncirin, nobiletin and tangeretin all exhibited an improvement effect on glycolipid metabolism disorder (19–23), while naringin, hesperidin, nobiletin and tangeretin have been found to regulate the gut microbiome (19, 23–25). It is worth noting that hesperidin is a component that should be detected in the drug standard of CRP extract, suggesting that hesperidin may play a key role in this process (**Supplementary Materials**: Drug standard of CRP extract). However, further researches are required to explore the content of each component in CRP extract and the role of each component. In summary, these fingding indicated that CRP extract may improve the gut microbiome dysbiosis in childhood, thereby improving HFD-induced glycolipid metabolism disorder in adulthood.

In order to further understand the mechanism underlying the effects of the CRP extract, 16S rRNA sequencing technology was used to assess the changes of gut microbiome in the mice in each group. Bacteroidetes and Firmicutes are the two dominant bacteria in the gut, and many studies have demonstrated that the relative abundance of Firmicutes is increased while the relative abundance of Bacteroides is decreased in glycolipid metabolism disorder (26–28). Parabacteroides belong to the Porphyromonadaceae family and have been found to be more dominant in obese subjects with low relative abundance (29). Short-chain fatty acids and bile acids are the main metabolites of Parabacteroides and studies have shown that the relative abundance of Parabacteroides is reduced in mice with glycolipid metabolism disorder induced by an HFD (30). Adlercreutzia is a genus from the phylum Actinobacteria. This genus was originally identified in human faeces and was found to play an important role in glycolipid metabolism (31). Studies have indicated that there is low abundance of Adlercreutzia in mice with glycolipid metabolism disorder (32). Prevotella and Paraprevotella can degrade carbohydrates and polysaccharides in food and participate in the synthesis of vitamins in the body. Studies have shown that the abundance of Prevotella and Paraprevotella are negatively correlated with serum TG, TC and LDL-C levels, and positively correlated with the HDL-C level, suggesting that Prevotella and Paraprevotella may have anti-obesity effects (33). Sutterella belongs to the family Sutterellaceae and has been shown to be associated with liver lipogenesis; studies have shown that the relative abundance of Sutterella is increased in obese individuals (34). Bilophila is an LPS-producing bacteria that can aggravate inflammation in HFD-mice and cause metabolic disorder. Studies have shown that mice with glycolipid metabolism disorder have lower relative abundance of Bilophila (35). Bifidobacterium is a short-chain fatty acids (SCFAs)-producing bacteria that can degrade polysaccharides and dietary fibre. The relative abundance of Bifidobacterium is directly related to improved body weight and glycolipid metabolism (36). Veillonella has been shown to be related to lactate metabolism, and Veillonella is positively correlated with glycolipid metabolism (37). Butyricimonas is a beneficial bacteria that can produce SCFA to reduce inflammation. Studies have shown that increased relative abundance of Butyricimonas is associated with improved metabolic parameters and insulin resistance in mice (38). Taken together, these results indicate that CRP extract improved the gut microbiome in AZT-treated juvenile mice and improved glycolipid metabolism disorder in adulthood under HFD feeding.

The heat map indicated that the relative abundance of Enterococcus and Streptococcus in the AZT group exhibited an increasing trend, while there was a decreasing trend in the CRP group. Enterococcus is one of the normal gut microbiome present in humans and animals. It was previously thought to be harmless to the body, but its pathogenicity has been demonstrated in recent years. Studies have indicated that Enterococcus increases obesity and causes insulin resistance (39). Most Streptococcus are conditional pathogens that can cause body infections such as sepsis and endocarditis. Studies have shown that the relative abundance of Streptococcus is increased in obese individuals (40). Enterococcus and Streptococcus can metabolize choline substances into trimethylamine (TMA) through microbial enzyme complexes, finally increasing the serum level of TMAO (41). Therefore, the TMAO levels in serum were examined in this study. The results showed that serum TMAO levels were increased in the AZT group and decreased in the CRP group. Glycolipid metabolism disorder is often accompanied by low-grade chronic inflammation (42), and the NLRP3/caspase-1 signalling pathway plays an important role in the development of inflammation in glycolipid metabolism disorder (43). Studies indicate that TMAO may cause glycolipid metabolism disorder by increasing NLRP3/caspase-1-mediated inflammation (10, 44). Therefore, inflammation levels and NLRP3/caspase-1 signalling pathway-related proteins were examined in this study. The results demonstrated that serum inflammation markers and NLRP3/caspase-1 signalling pathway-related proteins were increased in the AZT group and decreased in the CRP group.

There are several strengths and limitations of this research that should be noted. The strengths of this study are as follows: 1) There were no adverse reactions due to the CRP intervention observed in this study. 2) In order to reduce the irritation to animals, the antibiotics were dissolved in water to allow the mice to drink freely, instead of administering them by gavage. 3) The estimated required sample size based on the degrees of freedom for analysis of variance was five in each group, but due to the large individual differences in the detection of gut microbiome, the sample size of each group was increased to ten in each group. The limitations of this study are as follows: 1). There was only one intervention dose of CRP; thus, it was not possible to determine the dose-effect relationship between CRP and changes in gut microbiome. However, the dose used in this study is based on the body surface area conversion of humans and mice, so its efficacy is worthy of affirmation. 2) Only TMAO, which is a metabolite of gut microbiome, was tested; the role of other metabolites in this process was not able to be determined. In this study, the results suggest that the changes of gut microbiome is closely related to TMAO, so it is reason to believe that TMAO may play a key role in this process. Therefore, there is no doubt about the important role of TMAO. 3) The content of each component of CRP was not quantitatively analysed to identify the effective components that play key roles. An important reason is that we wonder to know whether the detection components of CRP extract are comparable to the drug standard. 4) For animal models, we should set up more groups to explore the effects of AZT and CRP treatments on the gut microbiome under the ND. But our research is sufficient to show whether early-life AZT exposure in mice could promote HFD-induced glycolipid metabolism disorder in adulthood and the intervention of CRP extract in the process. Overall, this research indicates that early-life AZT exposure in mice promotes HFD-induced glycolipid metabolism disorder in adulthood, and CRP extract can improve this glycolipid metabolism disorder by regulating AZT-induced gut microbial disorder in mice.

## Conclusion

In conclusion, the results showed that early-life AZT exposure increases the susceptibility to HFD-induced glycolipid metabolism disorder in adult mice, and CRP extract can decrease the susceptibility to glycolipid metabolism disorder in mice by regulating gut microbiome. These findings provide information about the health benefits of CRP and verify the potential of CRP as an effective intervention for the prevention of antibiotic-associated glycolipid metabolism disorder.

## Data Availability Statement

The data supporting the conclusions of this article is included within the article. Data of microbiota assessment has been deposited in SRA database (SRA accession: PRJNA752871).

## Ethics Statement

The animal study was reviewed and approved by Animal Ethical Committee of Guangzhou Institute of Sport Science, Guangzhou, Guangdong, China (Ethics certificate number: GZTKSGNX-2016-3).

## Author Contributions

XSZ and LL conceived, designed, and supervised the study. HL and YY performed the animals experiment before the mouse sacrifice. HL, XHZ, and QH performed the detection of related indicators when mice were sacrificed. YY, MW, and LZ performed data collation and analysis. HL, XS, LC, and YL wrote the manuscript. PJ, JD, XF, and HK modified the manuscript. All authors contributed to the article and approved the submitted version.

## Funding

This work was supported by the National Key R&D Program of China (2020YFC2003100, 2020YFC2003101), the Key Project of National Natural Science Foundation of China (81830117), the National Science Foundation of China (81774212), the Natural Science Foundation of Guangdong Province, China (2018A030313320,2018A030313375,2019A1515010400), the Science & Technical Plan of Guangzhou, Guangdong, China (201903010069), and the Innovation Team and Talents Cultivation Program of National Administration of Traditional Chinese Medicine. (No: ZYYCXTD-C-202001) (ZYYCXTD-C-202001) and the National Traditional Chinese Medicine Administration National Traditional Chinese Medicine Experts Inheritance Studio Construction Project [Ministry of Medicine in China, No. (2019)41].

## Conflict of Interest

The authors declare that the research was conducted in the absence of any commercial or financial relationships that could be construed as a potential conflict of interest.

## Publisher’s Note

All claims expressed in this article are solely those of the authors and do not necessarily represent those of their affiliated organizations, or those of the publisher, the editors and the reviewers. Any product that may be evaluated in this article, or claim that may be made by its manufacturer, is not guaranteed or endorsed by the publisher.

## Abbreviations

CRP, Citrus reticulata pericarpium; AZT, azithromycin; HFD, high-fat diet; Ctrl, control; TMAO, trimethylamine N-oxide; TNF-α, tumor necrosis factor-α; IL-6, interleukin-6; IL-1β, interleukin-1β; caspase-1, cysteinyl aspartate specific proteinase 1; NLRP3, NOD-like receptor protein 3; TG, triglycerides; TC, total cholesterol; LDL-C, low density lipoprotein-cholesterol; HDL-C, high density lipoprotein-cholesterol; ND, normal diet; OGTT, oral glucose tolerance test.
