# Supplementary material for: Citrus reticulatae pericarpium Extract Decreases the Susceptibility to HFD-Induced Glycolipid Metabolism Disorder in Mice Exposed to Azithromycin in Early Life
Source: Front Immunol. 2021 Nov 19;12:774433. doi: 10.3389/fimmu.2021.774433 (PMC8640250; doi:10.3389/fimmu.2021.774433)
Supplement: Supplementary file 1 [file DataSheet_1.docx]

Supplementary Material

| **Table S1 Feed formulas of normal diet and high-fat diet** | | | | | | |  |
| --- | --- | --- | --- | --- | --- | --- | --- |
| Product | | | D12450B | | D12492 | |  |
|  |  |  | gm% | kcal% | gm% | kcal% |  |
|  |  |  |  |  |  |  |  |
| Casein | | | 200 | 800 | 200 | 800 |  |
| L-Cystine | | | 3 | 12 | 3 | 12 |  |
| Com Starch | | | 315 | 1260 | 0 | 0 |  |
| Maltodextrin | | | 35 | 140 | 125 | 500 |  |
| Sucrose | | | 350 | 1400 | 68.8 | 275.2 |  |
| Cellulose | | | 50 | 0 | 50 | 0 |  |
| Soybean Oil | | | 25 | 225 | 25 | 225 |  |
| Lard | | | 20 | 180 | 245 | 2205 |  |
| Mineral Mix S10026 | | | 10 | 0 | 10 | 0 |  |
| DiCalcium Phosphate | | | 13 | 0 | 13 | 0 |  |
| Calcium Carbonate | | | 5.5 | 0 | 5.5 | 0 |  |
| Potassium Citrate 1H2O | | | 16.5 | 0 | 16.5 | 0 |  |
| Vitamin Mix 93G | | | 10 | 40 | 10 | 40 |  |
| Choline Bitartrate | | | 2 | 0 | 2 | 0 |  |
| Yellow | | | 0.05 | 0 | 0.04 | 0 |  |
| Red Dye | | | 0 | 0 | 0 | 0 |  |
| Blue Dye | | | 0 | 0 | 0.01 | 0 |  |
| Total | | | 1055.05 | 4057 | 773.85 | 4057.2 |  |
| **Table S2 Retention times of ten components in CRP extract** | | | | | | | |
| NO. | retention times (min) | Components | | | | | |
| 1 | 7.33 | naringin-4’-O-glucoside | | | | | |
| 2 | 12.73 | narirutin | | | | | |
| 3 | 13.2 | Naringin | | | | | |
| 4 | 13.83 | hesperidin | | | | | |
| 5 | 17.63 | poncirin | | | | | |
| 6 | 25.37 | nobiletin | | | | | |
| 7 | 27.32 | tangeretin | | | | | |

**
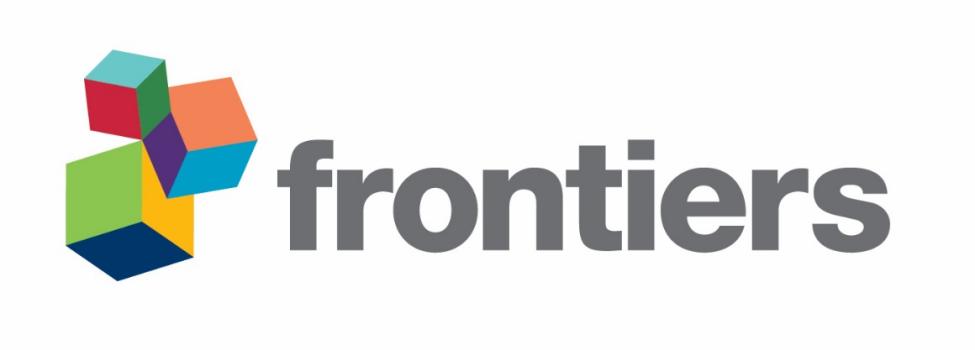
**
